# Rapid Detection of Sugar

**Published:** 1855-11

**Authors:** 


Rapid Detection of Sugar.—M. Botte has several times employed the method suggested by Liebig of quickly detecting sugar. A small quantity of ox-gall is dissolved in the suspected fluid in a test-glass, and a quantity of concentrated sulphuric acid equal in amount to that of the fluid in the glass is rapidly added, care being taken to pour it along the side of the glass. If sugar is present a beautiful purpurine is immediately produced.—(*Rev. Médicale*, June: p. 685.) *Ibid.*

